# Who have a better-kidney-related quality of life: peritoneal dialysis or hemodialysis patients? A cross sectional study from Saudi Arabia

**DOI:** 10.1186/s12882-023-03270-7

**Published:** 2023-07-20

**Authors:** Mohammed Alshehri, Abdullah Alshehri, Ali Alfageeh, Khalid Asiri, Alwaleed Alshehri, Feras Alqahtani, Mazen Alshehri, Mohanad Alshabab, Omar Asiri

**Affiliations:** 1 Nephrology Section, Internal Medicine Department, College of Medicine, King Khalid University, Abha, 61421 Saudi Arabia; 2 Senior Health Expert, Public Health Authority, Riyadh, 13351 Saudi Arabia; 3 Nephrology Center, Armed Forces Hospital of Southern Region, 101, Khamis Mushayte, Saudi Arabia; 4 Nephrology Section, Internal Medicine Department, King Khalid University Medical City, Abha, 61421 Saudi Arabia; 5 Medical Intern at College Of Medicine, King Khalid University, Abha, 61421 Saudi Arabia; 6 Medical Student at College Of Medicine, King Khalid University, Abha, 61421 Saudi Arabia

**Keywords:** End stage kidney disease, Hemodialysis, Peritoneal dialysis, Quality of life

## Abstract

**Background:**

End-stage kidney disease (ESKD) substantially impacts different aspects of patients' lives, including mental and physical health, and overall quality of life. The aim of our study is to assess the quality of life (QoL) and the associated predictors in ESKD Saudi Arabian patients receiving either hemodialysis (HD) or peritoneal dialysis (PD). Saudi Ministry of health (MOH) is advancing dialysis care in the country with more focus on patients’ satisfaction and QoL. However, the data regarding QoL in Saudi Arabian dialysis patients is limited.

**Methods:**

A cross sectional study was carried out using Kidney Disease Quality of Life scale (KDQOL-36) to assess the QoL. We disturbed a validated formal arabic version of the questionnaire. Patients older than 18-year-old and attending dialysis clinics in Aseer region, Saudi Arabia, were invited to participate in the study.

**Results:**

A total of 152 responses were analyzed, which were separated into two primary groups: the HD group (98 patients) and the PD group (54 patients). Our results showed no significant differences between the two groups except for the KDQOL-SF-36 physical composite score at which the PD group had a higher mean than the HD group 44.75 vs 37.84, respectively (*p* < 0.001). However, more PD patients reported feeling depressed compared to HD patients.

**Conclusions:**

Kidney Disease Quality of Life scale scores were comparable between HD and PD groups except for the physical composite score. On the other hand, PD patients tend to suffer from depression more than HD patients. Interventions to attenuate the physical deconditioning and depressive symptoms in HD and PD patients, respectively, are crucial. Future prospective studies with larger sample sizes are warranted.

**Supplementary Information:**

The online version contains supplementary material available at 10.1186/s12882-023-03270-7.

## Introduction

End-stage kidney disease (ESKD) is increasing worldwide [1, 2]. It substantially impacts different aspects of patients' lives, including mental health [3], physical health, and overall quality of life [4]. ESKD is treated with hemodialysis (HD), peritoneal dialysis (PD), or kidney transplantation. Among these, HD remains the most common treatment. According to the Saudi Center for organ transplantation (SCOT) 2019 report [5], the total number of ESKD patients in Saudi Arabia is 28,256. The majority of these patients (19,522) or 69% were on HD, a quarter of them were post-transplant patients (7,188), and the minority of them were on PD (1,546) or (7%) [6]. The percentage of ESKD patients on PD varies according to the centers and the national region of interest. For instant, the total ESKD patients in the southern region, where our study is performed, is close to 3000 patients, however only 4% are on PD, which is the lowest percentage in Saudi Arabia according to the report. In general, PD patients in Saudi Arabia account for 7% of the total ESKD patients. This number is close to the reported data from unites states [7]. However, it is extremely low compared to Australia, Canada, Hong Kong, and Mexico [8]. Hence, the utilization of PD in Saudi Arabia remains suboptimal. Lack of pre-dialysis education, patients' and nephrologists' perspective on PD, and the deficiency of established PD programs are major factors behind the underutilization of PD in Saudi Arabia [9].

In 2019, Saudi Arabia had a total of 21,068 dialysis patients, of which 19,522 received HD and 1,546 received PD. During the same year, there were 4,740 new patients who required dialysis initiation. The country had 278 HD centers with 8,165 dialysis machines to meet the growing demand. HD was provided to 36% of patients through the Ministry of Health (MOH) outsourcing programs and 27% through MOH hospitals. There were 1,121 kidney transplantation surgeries performed in Saudi Arabia. Table 1 provides more detailed information on the number of dialysis patients, HD centers, and kidney transplantation surgeries in Saudi Arabia.

**Table 1:** General info of the end stage kidney disease situation in Saudi Arabia and the provided services (2019)^a^

**Overview of the Hemodialysis and Peritoneal Dialysis Services**					
Total Number of Hemodialysis Centers			278		
Total Number of Hemodialysis Machines			8165		
Total Number of Hemodialysis Patients			19,522		
Total Number of Peritoneal Dialysis Patients			1546		
**Incidence and Prevalence of Dialysis Patients According to Region in 2019**					
**Region**	**Population 2019**	**Dialysis Patients 2019**	**Prevalence (pmp)**	**New Patients**	**Incidence (pmp)**
Western	10,991,683	6,821	621	1,574	143
Central	9,902,559	6,597	666	1,635	165
Eastern	5,028,753	2,743	545	618	123
Southern	4,948,090	3,108	628	584	118
Northern	2,542,575	1,799	708	329	129
**Total**	**33,413,660**	**21,068**	**631**	**4,740**	**142**
**Distribution of Chronic Hemodialysis Patients by Dialysis Sector**					
**Sector**			**Number of Centers**	**Number of Patients**	
MOH Outsourcing Dialysis Program			62	7,076 (36%)	
MOH			132	5,258 (27%)	
Governmental Non-MOH			24	2,994 (16%)	
Private & Charitable hospitals			54	2,987 (15%)	
King Abdullah Hemodialysis Projects			6	1,207 (6%)	
**Total**			**278**	**19,522 (100%)**	
**Kidney Transplantations in Saudi Arabia, 2019**					
Living Donors			981		
Deceased Donors			140		
**Total**			**1,121**		

*MOH* Ministry of health, *PMP* Per million population

^a^Annual report for organ transplantation in Kingdom of Saudi Arabia, 2019 [5]

One of the main differences between the two dialysis modalities is that PD is usually delivered at home and gives the patients more independence and self-control over their treatment and time, while in-center HD patients must commute to the dialysis centers several times a week with tight timing and scheduling. On the other hand, PD requires a robust support system including family support to help managing PD materials and equipment [10]. These factors, among others, would likely influence the patient's QoL and might help guiding the modality selection by the patients and the clinicians.

Several tools have been used to assess the impacts of dialysis on the quality of life in this population such as the Kidney Disease Quality of Life (KDQOL) and its short form. Recently, Elamin, et al. published the first formal arabic validated version of the KDQOL-36 [11]. They used the formal arabic translation compared to the previous versions where non universal arabic dialects were used [12]. Previous studies reported that the quality of life in patients undergoing PD is generally better comparing to HD patients [13]. However, the data from Saudi Arabia is scarce and limited to one study from 2012 by Al Wakeel, et al. [14]. The aim of our study is to assess the QoL and the associated predictors in ESKD Saudi Arabian patients receiving either HD or PD, by using the formal Arabic translation of KDQOL-36. Moreover, we will compare the findings to the previous national and international reports.

## Materials and methods

To conduct this Quality-of-Life study, we followed the guidelines of prior studies and used a comparative cross-sectional design [15, 16]. In addition to the standard demographic questions including name, age, and length of time on dialysis, the questionnaire also included the Kidney Disease Quality of Life scale (KDQOL-36). The KDQOL scale is disease-specific and was developed to address the unique health concerns of patients with kidney disease and in particular dialysis patients, regarding several parameters such as the burden of kidney disease, employment and mental health, quality of social and intimate relationships, as well as the impact of dialysis on patients' sense of empowerment and hope. Its 36 items are divided into six groups, overall wellness, physical and mental health, social support, the effect of sickness on daily life, and how satisfied the patients are with their healthcare in terms of cost and quality. The responses from the instrument were scored in accordance with the criteria established by the KDQOL-SF [17]. This questionnaire was reprinted with permission from the RAND© Corporation. RAND's permission to reproduce the survey is not an endorsement of the products, services, or other uses in which the survey appears or is applied. An arabic version of the questionnaire was used after we obtained the permission from the authors [11].

All patients older than 18-year-old attending dialysis clinics from January 2022 to December 2022 were invited to participate in the study. The study included 152 ESKD patients from Aseer Region, Saudi Arabia. We included patients from Ministry of Health (MOH), Armed forces hospital, and private dialysis units. Patients who refused to participate, younger than 18-year-old, and patients who had cognitive impairment that affect their answers were excluded from the study. We also excluded the patients who had been on dialysis for less than 3 months.

We compared two groups of patients receiving different types of dialysis treatments, one group consisted of 98 HD patients and the other group consisted of 54 PD patients. Before conducting any interviews, all the patients provided informed written consents. The researchers conducted individual interviews with each participant and then gave them a questionnaire to fill out. This study's protocol was approved by the Institutional Review Board at the Aseer Directorate of Health Affairs in Abha, Saudi Arabia (REC-16–01-022).

To analyze the data, we used SPSS version 29.0 for data entry and statistical analysis, which is a software package from IBM Corp located in Armonk, New York, United States. We used the chi-square test to assess the significant association between categorical data. If the chi-square assumptions were not met, we used the Two Sample Z test of proportions to compare proportions between two groups. For continuous data, we used the Kolmogorov–Smirnov test of normality to test normality. If the means of two groups for normally distributed continuous data were compared, we used independent samples t-test to compare the means. However, if the data did not follow a normal distribution, we used non-parametric tests like the Mann–Whitney U test. A *P* value of 0.05 was taken as an indicator of statistical significance.

## Results

A total of 152 responses were analyzed, which were separated into two primary groups: the HD group, which included 98 respondents, and the PD group, which included 54 respondents. All the HD patients were on in-center HD while all the PD were on automated cyclic PD (APD). Table 2 shows the demographic characteristics of the two groups.

**Table 2:** Demographic characteristics

*n* = 152	Hemodialysis = 98	Peritoneal Dialysis = 54	*P*-value
**Age**			
Less than 40	20 (20.4%)	33 (61.11%)	
40—60	46 (46.94%)	16 (29.63%)	
More than 60	32 (32.66%)	5 (9.26%)	< 0.001* ^a^
**Gender**			
Male	60 (61.22%)	29 (53.7%)	
Female	38 (38.78%)	25 (46.3%)	0.368 ^a^
**Marital Status**			
Married	91 (92.86%)	32 (59.26%)	
Single	7 (7.14%)	22 (40.74%)	< 0.001* ^a^
**Income**			
Less than 5000 SAR	58 (59.19%)	31 (57.31%)	0.8299 ^b^
5000 – 10,000 SAR	31 (31.63%)	18 (33.33%)	0.8306 ^b^
More than 10,000 SAR	9 (9.18%)	5 (9.26%)	0.9838 ^b^
**Employment Status**			
Unemployed	76 (77.55%)	37 (68.52%)	
Employed	22 (22.45%)	17 (31.84%)	0.222 ^a^
**Education**			
Did not attend school	26 (26.53%)	6 (11.11%)	
Elementary school	33 (33.67%)	15 (27.78%)	
High School	25 (25.51%)	11 (20.37%)	
Higher Education	14 (14.29%)	22 (40.74%)	0.002* ^a^
**Who support you on dialysis?** **(Self-dependent – other)**			
Self-Dependent	50 (51.02%)	24 (44.44%)	
Other	48 (48.98%)	30 (55.56%)	0.438 ^a^
**Duration of dialysis (in years)**			
Less than a year	15 (15.31%)	23 (42.59%)	
1—3	31 (31.63%)	15 (27.78%)	
More than 3 years	52 (53.06%)	16 (29.63%)	< 0.001* ^a^
**Chronic Medical Condition**			
Hypertension	66 (67.35%)	43 (79.63%)	0.0899 ^b^
Cardiac disease †	19 (19.39%)	10 (18.52%)	0.7310 ^b^
Diabetes Mellitus	45 (45.92%)	16 (29.63%)	0.0505 ^b^
Others	42 (42.86%)	14 (25.93%)	0.0382* ^b^

*n* Sample size

^a^*P*-value for chi-square test

^b^*P*-value for comparing 2 proportions

^*^Significant difference

^†^heart failure or coronary artery disease

Most of the PD respondents were under 40 years of age, while most of HD patients were over 40 years of age. The response rate was higher among PD patients compared to HD patients, 36% and 25%, respectively. The HD group had a higher prevalence of married patients than the PD group. Most of the HD patients (84%) received dialysis for more than 3 years compared to (57%) of the PD patients. The education level differed between the two groups; the PD respondents tended to have a higher education degree, while the HD respondents tended to have not attended school or had an elementary school education only. Both groups had nearly a similar proportion of gender and employment status. The HD group had a higher prevalence of diabetes and other comorbidities and nearly a similar prevalence of cardiac diseases; however, the PD group had a higher prevalence of hypertension.

The KDQOL-SF questionnaire consists of 36 ordinal scale questions aimed to assess the quality of life among the respondents. The questionnaire has five areas for scoring: symptom/problem list (I1-I12), effects of kidney disease, burden of kidney disease, SF-12 Physical Composite, and SF-12 Mental Composite. The study found a significant difference between the two dialysis groups in 8 individual items of the questionnaire (items 3, 5, 8, 11, 13, 19, 26, and 31) Table 3. The PD group had better scores in 7 out of the 8 items (items 3, 5, 8, 13, 19, 26, and 31), while the HD group had a better score on (item 11).

**Table 3:** Comparing questionnaire

*n* = 152	*p*-value	Who had a better score?
Item 3: Does your health now limit you in some activities like climbing several flights of stairs?	0.020	PD
Item 5: During the past 4 weeks, were you limited in the kind of work or other activities in your work or other regular daily activities as a result of your physical health?	0.005	PD
Item 8: During the past 4 weeks, how much did pain interfere with your normal work (including both work outside the home and housework)?	< 0.001	PD
Item 11: How much of the time during the past 4 weeks, have you felt downhearted and blue?	0.026	HD
Item 13: My kidney disease interferes too much with my life	0.031	PD
Item 19: During the past 4 weeks, to what extent were you bothered by cramps?	0.016	PD
Item 26: During the past 4 weeks, to what extent were you bothered by numbness in hands or feet?	< 0.001	PD
Item 31: How much does kidney disease bother you in your ability to work around the house?	0.018	PD

*n* Sample size

• Comparison done using Mann–Whitney U test

• The rest of the questionnaire items can be found in Supplementary files

Regarding the overall scores, our results showed no significant differences between the two groups except for the SF-12 Physical Composite score at which the PD group had a higher mean than the HD group (Table 4). The different on physical composite score between the two groups remained significant even after performing subgroup analysis regarding dialysis duration for less than 3 years; the HD group had 46 respondents, while the PD group had 38 respondents. The overall scores were calculated using a spreadsheet developed by the RAND Corporation© [18].

**Table 4:** Comparing scores between hemodialysis and peritoneal dialysis

*n* = 152	Hemodialysis = 98 Mean (SD)	Peritoneal Dialysis = 54 Mean (SD)	*p*-value
Symptom/ problem list (I1-I12)	64.63 (21.91)	70.8 (18.98)	0.082^a^
Effects of kidney disease on the daily life	64.19 (23.52)	67.48 (18.93)	0.380^a^
Burden of kidney disease	48.66 [Median: 50.00] (25.98)	47.45 [Median: 46.88] (24.81)	0.657^b^
SF-12 Physical Composite #	37.84 [Median: 37.17] (10.21)	44.75 [Median: 41.69] (9.60)	< 0.001*^b^
SF-12 Mental Composite #	45.03 (11.05)	43.07 (10.47)	0.287^a^

*n* Sample size

Normality was tested using Kolmogorov–Smirnov test of normality

^a^Compared using independent samples t-test to compare 2 means

^b^Compared using Mann–Whitney U test

* Significant difference

## Discussion

Quality of life was assessed using KDQOL-SF-36. This tool is designed for dialysis patients, and it has been translated to more than 30 languages. However, first formal arabic validated translation was published recently [11]. Compared to the previous published arabic version, this version used the formal arabic dialect “Fusha”, which is the common dialect among people in different parts of the Arab world. The previous version used the Egyptian dialect, which is not always easy to understand to other arabic speakers [19].

Using this tool, we found that PD patients are able to perform more physical activity and suffer less from pain, cramps, and numbness. The HD patients reported a great impact of the burden of the disease on their life compared to the PD patients. However, HD patients were less likely to have symptoms of depression compared to the PD patients.

Being older and on dialysis for a longer period could explain why HD patients score less on physical activity related items. The age gap between HD and PD patients has been observed in previous epidemiological studies [20, 21]. The shorter time on dialysis for PD patients is likely explained by the fact that most of PD patients are not on PD after a few years due to either technique failure, transitioning to HD or transplant, or death [22]. Moreover, it is known that the longer the duration of PD is, the higher is the risk of transitioning to HD [23]. These differences, collectively, can be explained by the possibility that PD programs generally select younger, more educated, and healthier patients [24, 25], which may have contributed to the lower response rate observed among HD patients.

The process of commuting to dialysis center, experiencing the dialysis canulation pain, ultrafiltration adjustment, and hemodynamic changes with HD may be the reasons behind the differences on disease burden, pain, cramps, and numbness that were reported more by HD patients. On the contrary, less depressive symptoms in HD patients compared to PD patients is probably due to the social interactions associated with going to the HD centers. This finding is consistent with a previous cross sectional study from Saudi Arabia [26]. Depression is highly prevalent among ESKD population in general, with up to 40% of patients may have depression. Nonetheless, the data is not consistent regarding the effect of dialysis modality on the depression prevalence [27]. PD patients have the privileges of more control on their treatments, more independent, and lack of the pain related to pricking for access or with other HD related procedures. However, they lack the social interactions with medical staff and other patients in the dialysis centers. This, in part, may explain why more PD patients felt more depressed.

The two groups' overall QoL scores were similar except for the physical composite (Table 4). Our findings differ from Al wakleel et al. study [14], where PD patients had higher scores in all domains except physical composite. We proposed several causes explaining the difference between the two studies. First, the previous study was conducted between 2007–2008. PD technology has developed substantially since then. Second, all the patients in our study were on APD. Third, our PD patients are younger compared to the previous study. Fourth, the dialysis durations significantly differed between the two groups, shorter in PD patients. For this purpose, we did a subgroup analysis regarding time on dialysis to examine the effect of duration on physical activity. The difference remains significant between the two groups. Due to the low sample size, we could not do a subgroup analysis regarding age or other demographic factors. Lastly, the Saudi MOH developed the dialysis outsourcing program in 2013. Through this program, MOH contracted multiple international hemodialysis companies, including DaVita from the United States and Diaverum from Sweden [28] to take over the care of HD patients. The quality of delivered care improved substantially with strict MOH implanted regulations and policies. These new regulations may have affected our findings and showed comparable QoL between HD and PD patients compared to previous national and international studies. Of note, PD was introduced early to Saudi Arabia, but it is currently only offered by large hospitals in main cities. There is a significant knowledge gap among both nephrologists and patients in Saudi Arabia regarding PD [28]. Education, promotion, and initiation of a "PD first" program at a national level could accelerate the growth of PD in the country.

Our study has several limitations. 1) The insignificant differences between the two groups could have been significant if the sample size had been larger. However, the sample may still be representative since we included more than a third of the PD patients in the region. HD patients, on the other hand, were from all the centers in the region. 2) Our study does not include laboratory data, like anemia or nutritional status. 3) Our study is cross-sectional, and a causal relationship cannot be established.

## Conclusion

KDQOL-36 scores were comparable between HD and PD groups except for the physical composite score. PD patients, on the other hand, tend to suffer from depression more than HD patients. Multidisciplinary interventions involving policymakers, dialysis units directors, nephrologists, physical therapists, and social workers are crucial to attenuate the impacts of ESRD patients on all QoL aspects. Physical deconditioning and depressive symptoms are more specific to HD and PD patients, respectively. Future prospective studies with larger sample sizes are warranted.

## Supplementary Information

**Additional file 1. **The KDQOL-36™ Survey.

**Additional file 2: Table 1S.** Comparing Questionnaire. **Table 2S.** Questionnaire Items.

## Data Availability

The datasets generated and/or analyzed during the current study are available from the corresponding author on reasonable request.

## Abbreviation

ESKD: End-stage kidney disease
QoL: Quality of life
HD: Hemodialysis
PD: Peritoneal dialysis
MOH: Ministry of health
PMP: Per million population
KDQOL: Kidney Disease Quality of Life
SCOT: Saudi Center for organ transplantation
APD: Automated peritoneal dialysis
