# Supplementary material for: Who have a better-kidney-related quality of life: peritoneal dialysis or hemodialysis patients? A cross sectional study from Saudi Arabia
Source: BMC Nephrol. 2023 Jul 20;24:216. doi: 10.1186/s12882-023-03270-7 (PMC10360311; doi:10.1186/s12882-023-03270-7)
Supplement: Supplementary file 2 — Additional file 2: Table 1S. Comparing Questionnaire. Table 2S. Questionnaire Items. [file 12882_2023_3270_MOESM2_ESM.docx]

|  | Table 1S: Comparing Questionnaire | | |  |
| --- | --- | --- | --- | --- |
| n = 152 | | p-value | Who had a better score? | |
| 1. Item 1 | | 0.316 | ----- | |
| 1. Item 2 | | 0.16 | ----- | |
| 1. Item 3 | | 0.020 | PD | |
| 1. Item 4 | | 0.055 | ----- | |
| 1. Item 5 | | 0.005 | PD | |
| 1. Item 6 | | 0.719 | ----- | |
| 1. Item 7 | | 0.904 | ----- | |
| 1. Item 8 | | <0.001 | PD | |
| 1. Item 9 | | 0.932 | ----- | |
| 1. Item 10 | | 0.06 | ----- | |
| 1. Item 11 | | 0.026 | HD | |
| 1. Item 12 | | 0.606 | ----- | |
| 1. Item 13 | | 0.031 | PD | |
| 1. Item 14 | | 0.696 | ----- | |
| 1. Item 15 | | 0.375 | ----- | |
| 1. Item 16 | | 0.057 | ----- | |
| 1. Item 17 | | 0.337 | ----- | |
| 1. Item 18 | | 0.259 | ----- | |
| Comparison done with Mann-Whitney U test | | | | |

|  | Table 1S: Comparing Questionnaire (continued) | | |  |
| --- | --- | --- | --- | --- |
| n = 152 | | p-value | Who had a better score? | |
| 1. Item 19 | | 0.016 | PD | |
| 1. Item 20 | | 0.288 | ----- | |
| 1. Item 21 | | 0.225 | ----- | |
| 1. Item 22 | | 0.059 | ----- | |
| 1. Item 23 | | 0.189 | ----- | |
| 1. Item 24 | | 0.307 | ----- | |
| 1. Item 25 | | 0.217 | ----- | |
| 1. Item 26 | | <0.001 | PD | |
| 1. Item 27 | | 0.111 | ----- | |
| 1. Item 28 | | 0.766 | ----- | |
| 1. Item 29 | | 0.596 | ----- | |
| 1. Item 30 | | 0.706 | ----- | |
| 1. Item 31 | | 0.018 | PD | |
| 1. Item 32 | | 0.528 | ----- | |
| 1. Item 33 | | 0.258 | ----- | |
| 1. Item 34 | | 0.252 | ----- | |
| 1. Item 35 | | 0.898 | ----- | |
| 1. Item 36 | | 0.964 | ----- | |
| Comparison done using Mann-Whitney U test | | | | |

|  | Table 2S: Questionnaire Items |  |
| --- | --- | --- |
| 1. In general, how would you describe your health? | | |
| 1. Does your health now limit you in some activities like moving furniture, cleaning house, moving inside the house? | | |
| 1. Does your health now limit you in some activities like climbing several flights of stairs? | | |
| 1. During the past 4 weeks, have you accomplished less than you would like in your work or other regular daily activities as a result of your physical health? | | |
| 1. During the past 4 weeks, were you limited in the kind of work or other activities in your work or other regular daily activities as a result of your physical health? | | |
| 1. During the past 4 weeks, have you accomplished less than you would like in your work or other regular daily activities as a result of your emotional problems? | | |
| 1. During the past 4 weeks, were you unable to do your work or other regular daily activities as carefully as usual as a result of your emotional problems? | | |
| 1. During the past 4 weeks, how much did pain interfere with your normal work (including both work outside the home and housework)? | | |
| 1. How much of the time during the past 4 weeks, have you felt calm and peaceful? | | |
| 1. How much of the time during the past 4 weeks, did you have a lot of energy? | | |
| 1. How much of the time during the past 4 weeks, have you felt downhearted and blue? | | |
| 1. During the past 4 weeks, to what extent has your physical health or emotional problems interfered with your normal social activities with family, friends, neighbors, or groups? | | |
| 1. My kidney disease interferes too much with my life | | |
| 1. Too much of my time is spent dealing with my kidney disease | | |
| 1. I feel frustrated dealing with my kidney disease | | |
| 1. I feel like a burden on my family | | |
| 1. During the past 4 weeks, to what extent were you bothered by soreness in your muscles? | | |
| 1. During the past 4 weeks, to what extent were you bothered by chest pain? | | |
| 1. During the past 4 weeks, to what extent were you bothered by cramps? | | |
| 1. During the past 4 weeks, to what extent were you bothered by itchy skin? | | |
| 1. During the past 4 weeks, to what extent were you bothered by dry skin? | | |
| 1. During the past 4 weeks, to what extent were you bothered by shortness of breath? | | |
| 1. During the past 4 weeks, to what extent were you bothered by faintness or dizziness? | | |
| 1. During the past 4 weeks, to what extent were you bothered by lack of appetite? | | |
| 1. During the past 4 weeks, to what extent were you bothered by feeling washed out or drained? | | |
| 1. During the past 4 weeks, to what extent were you bothered by numbness in hands or feet? | | |
| 1. During the past 4 weeks, to what extent were you bothered by nausea or upset stomach? | | |
| 1. During the past 4 weeks, to what extent were you bothered by problems with catheter or access site? | | |
| 1. How much does kidney disease bother you in fluid restriction? | | |
| Table 2S: Questionnaire Items (continued) | | |
| 1. How much does kidney disease bother you in dietary restriction? | | |
| 1. How much does kidney disease bother you in your ability to work around the house? | | |
| 1. How much does kidney disease bother you in your ability to travel? | | |
| 1. How much does kidney disease bother you in being dependent on doctors and other medical staff? | | |
| 1. How much does kidney disease bother you in stress or worries caused by kidney disease? | | |
| 1. How much does kidney disease bother you in your sex life? | | |
| 1. How much does kidney disease bother you in your personal appearance? | | |
